# An unusual presentation of an inflammatory fibroid polyp of the ileum: A case report

**DOI:** 10.3892/ol.2014.2674

**Published:** 2014-11-05

**Authors:** JUN SANG BAE, JI SOO SONG, SEUNG-MO HONG, WOO SUNG MOON

**Affiliations:** 1 Department of Pathology, Chonbuk National University, Medical School, Research Institute for Endocrine Sciences and Research Institute of Clinical Medicine, Chonbuk National University Hospital, Jeonju, Jeollabuk-do 561-756, Republic of Korea; 2 Department of Radiology, Chonbuk National University, Medical School, Research Institute for Endocrine Sciences and Research Institute of Clinical Medicine, Chonbuk National University Hospital, Jeonju, Jeollabuk-do 561-756, Republic of Korea; 3 Department of Pathology, Asan Medical Center, University of Ulsan College of Medicine, Seoul 138-736, Republic of Korea

**Keywords:** polyp, ileum

## Abstract

Inflammatory fibroid polyps (IFP) are rare, benign lesions of the gastrointestinal tract. Recent molecular studies of IFPs identified platelet-derived growth factor receptor α (PDGFRA)-activating mutations, suggesting possible neoplastic qualities to IFPs. IFPs originate from the submucosa and often extend into the overlying mucosa. Although certain IFPs infiltrate the muscularis propria focally, disruption of the muscularis propria and penetration into the subserosa is extremely rare. The current study presents an unusual case of an ileal IFP. A 48-year-old female visited the Department of Surgery, Chonbuk National University Hospital (Jeonju, Republic of Korea) due to abdominal pain. Radiological study demonstrated an ileocecal-type intussusception due to a luminal polypoid mass of the ileum. The excised tumor consisted of haphazardly arranged epithelioid and spindled cells in a fibromyxoid stroma, with an abundant vascular network, accompanied by an inflammatory reaction predominantly composed of eosinophilic infiltrates. The infiltrating tumor cells disrupted the muscularis mucosa above the tumor cells and the muscularis propria below the tumor cells, and extended into the subserosa. Immunohistochemically, the tumor cells were positive for vimentin and cluster of differentiation 34, while they were negative for keratin, PDGFRA, smooth muscle actin, desmin, S-100 protein, DOG-1 and c-kit. Sequencing analysis of c-kit exons 9, 11, 13 and 17, and PDGFRA exons 12 and 18 indicated a wild-type status. The patient has remained well for 12 months after surgery without further treatment, with no recurrence of the tumor. Although spread of IFP under the muscularis propria is rare, identification of similar cases and further study will enhance our understanding of the nature of this tumor.

## Introduction

Inflammatory fibroid polyps (IFPs) are rare, benign mesenchymal tumors that can occur throughout the gastrointestinal tract (1,2). The exact incidence of IFPs remains unclear. The tumor occurs most frequently in the stomach of adults and usually presents as a polypoid mass with a size of 1–5 cm. Although the majority of patients have a nonspecific presentation, those with small intestine lesions more commonly form a symptomatic mass causing obstruction by intussusception (1–5). IFPs are generally considered to be benign tumors with no malignant potential, and therefore local excision is an adequate treatment. Small, pedunculated IFPs may be successfully removed by endoscopic submucosal dissection (1–5). Mutations in platelet-derived growth factor receptor α (PDGFRA; chromosome 4q12) have been indicated to be involved in the development of this tumor (2–5). IFPs are submucosa-based lesions that frequently extend into the overlying mucosa; however, they rarely spread under the muscularis propria (1,2,6). The current study presents an unusual case of a PDGFRA wild-type ileal IFP that extended into the subserosal layer. Written informed consent was obtained from the patient.

## Case report

**Figure 1:**
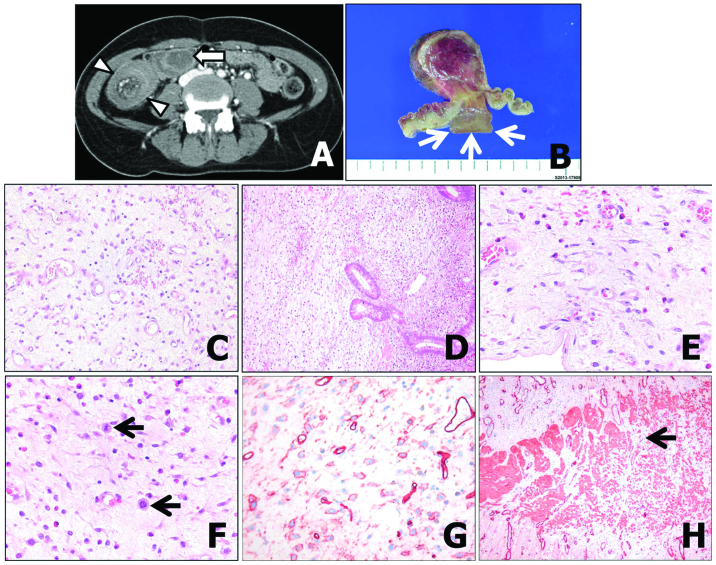
(A) Contrast-enhanced axial computed tomography image of a 48-year-old female patient with ileocecal intussusception. An ~3.5-cm oval-shaped mass can be observed as a lead point (arrow), and mesenteric fat and vessels, as well as bowel wall thickening of the intussusceptum and intussuscipiens (arrowhead) are also apparent. (B) The intraluminal polypoid mass extended into the subserosa (arrows). (C) The epithelioid tumor cells are embedded in an edematous, fibromyxoid stroma with prominent vasculature. The absence of concentric tumor cell proliferation should be noted. (stain, hematoxylin and eosin; magnification, ×200). (D) The tumor was centered in the submucosa and extended into the mucosa (magnification, ×100). (E) The spindle to epithelioid tumor cells were haphazardly arranged in the edematous subserosa. The heavy inflammatory infiltrate with eosinophils should be noted. (stain, hematoxylin and eosin; magnification, ×400). (F) Despite low tumor cellularity, the tumor cells showed mitotic figures (arrows) (stain, hematoxylin and eosin; magnification, ×400). (G) The tumor cells were positive for cluster of differentiation 34 (magnification, ×400). (H) Smooth muscle actin staining showed a disrupted muscularis propria by infiltrating tumor cells (arrow) (magnification, ×100).

A 48-year-old female visited the Department of Surgery, Chonbuk National University Hospital (Jeonju, Republic of Korea) for abdominal pain that had persisted for seven days. The results of routine hematological tests were within normal limits. A plain abdominal radiographical examination was unremarkable. The patient underwent contrast-enhanced abdominal computed tomography, which revealed an ileocecal-type intussusception in the right lower quadrant of the abdomen (Fig. 1A). There was an oval-shaped, 3.5-cm mass in the ileum, which was believed to be the lead point of intussusception. Segmental resection of the intussuscepted ileum was performed. The excised bowel segment revealed a 4.0×3.0-cm, luminal, polypoid mass that extended into the subserosa. The cut surface was myxoid and showed multiple hemorrhagic foci (Fig. 1B). Histologically, the tumor consisted of a mixture of bland-looking epithelioid, stellate and spindle cells, loosely distributed in a fibromyxoid stroma, with an abundant vascular network (Fig. 2A). The tumor cells were arranged in a random pattern; there was no so-called onion-like growth arrangement of the spindle cells around the glands and blood vessels. The tumor also revealed heavy eosinophilic infiltration with other inflammatory cells, including plasma cells, lymphocytes, histiocytes and mast cells. The majority of tumor cells were composed of epithelioid cells, and individual cells exhibited smooth, oval nuclei with modest eosinophilic cytoplasm. The infiltrating tumor cells disrupted the muscularis mucosa above the tumor cells and the muscularis propria below the tumor cells, and extended into the subserosa, suggesting a neoplastic nature of this lesion (Fig. 2B and C). There were 3–5 mitoses per 50 high-power fields (HPFs) (Fig. 2D). Immunohistochemically, the tumor cells were positive for vimentin and cluster of differentiation (CD)34, while they were negative for keratin, PDGFRA, smooth muscle actin, desmin, S-100 protein, DOG-1 and CD117 (c-kit) (Fig. 3A–C). The proliferation marker Ki-67 was positive in ~10% of the tumor cells (Fig. 3D). Sequencing analysis of c-kit exons 9, 11, 13 and 17, and PDGFRA exons 12 and 18 indicated a wild-type status. The patient has remained well for 12 months after surgery without treatment, with no recurrence of the tumor.

## Discussion

IFPs are rare mesenchymal lesions of the digestive system that were originally described by Vanek (7); they are considered reparative processes. Recent molecular studies of IFPs identified PDGFRA-activating mutations, suggesting a neoplastic nature to this lesion (2–5). Histologically, IPFs are characterized by proliferation of spindle, stellate or epithelioid cells accompanied by an inflammatory reaction, particularly eosinophilic granulocytes. The tumor cells are embedded in an edematous, fibromyxoid stroma, with prominent vasculature. A characteristic finding is the presence of concentric cuffing of the glands or vessels by spindle-shaped tumor cells. Concentric cuffing of tumor cells is more pronounced in gastric IFP than in intestinal IFP. IFPs tend to arise within the submucosa and often involve the mucosa (1,2,6).

In the present case, the IFP exhibited the following unusual features: Firstly, the tumor cells obliterated the muscularis propria and extended into the subserosa. IFPs are considered benign tumors without any risk for recurrence or metastasis following complete excision. The majority of IFPs are centered within the submucosa and rarely spread to the muscularis propria (1,2,6). A study by Makhlouf and Sobin demonstrated that among 45 reported cases of IFP, none extended into the serosa or subserosa tissue, although 7% of small intestinal IFPs infiltrated the muscularis propria (6). A search of the literature found only one similar case study describing a rectal IFP that exhibited attachment to the sacral bone, thus mimicking rectal cancer (8). Secondly, despite low tumor cellularity, the tumor cells demonstrated relatively high proliferative activity, as shown by Ki-67 labeling and mitotic figures. The majority of IFP cases have few or no mitotic figures, and Ki-67 labeling is <1% (5,6). In the study by Makhlouf and Sobin, only two out of the 45 IFPs showed 2–4 mitoses/50 HPFs, and no IFPs had more than 5 mitoses/50 HPFs (6). The present findings of subserosal extension of the lesion with disruption of the muscularis propria by infiltrating tumor cells and proliferative activity of the tumor cells supported a neoplastic nature of the current IFP. The mutation and activation of PDGFRA contribute to the development of IFPs (2–5). Huss *et al* reported that exon 12 PDGFRA mutations are more often associated with small intestinal IFPs, whereas exon 18 PDGFRA mutations occur frequently in gastric IFPs (5). A PDGFRA mutation or PDGFR expression was not identified in the present case. the reason for the lack of PDGFRA mutation for the current IFP is unclear. A possible explanation is a false-negative sequence analysis possibly resulting from the cellularity of these lesions, or the degradation of tumor DNA in the paraffin blocks, among other factors. This assumption is less likely in the present case, which showed no PDGFR expression, as none of the PDGFR-negative IFPs revealed PDGFRA mutations, although they showed typical histological features of IFPs (3). It has also been suggested that certain small intestinal IFPs are caused by molecular mechanisms other than PDGFRA activation (4). Indeed, previous studies showed that only 47 of 81 (58.0%) IFPs of the small intestine carried mutations in the PDGFR gene (2–5). It is difficult at present to elucidate the underlying mechanisms of this condition; further studies with a larger number of similar cases will be necessary to characterize the phenotype and nature of this tumor. After eight months of follow-up, the patient is in good health and no evident recurrence of the tumor has been detected.
